# Fluorescence background quenching as a means to increase Signal to Background ratio - a proof of concept during Nerve Imaging

**DOI:** 10.7150/thno.46806

**Published:** 2020-08-06

**Authors:** Tessa Buckle, Steffen van der Wal, Danny M. van Willigen, Germaine Aalderink, Gijs H. KleinJan, Fijs W.B. van Leeuwen

**Affiliations:** 1 Interventional Molecular Imaging Laboratory, Department of Radiology, Leiden University Medical Center, Leiden, The Netherlands.; 2 Department of Urology, Leiden University Medical Center, Leiden, The Netherlands.

**Keywords:** Pretargeting, fluorescence imaging, nerve imaging, image-guided surgery, fluorescence activation

## Abstract

**Introduction:** Adequate signal to background ratios are critical for the implementation of fluorescence-guided surgery technologies. While local tracer administrations help to reduce the chance of systemic side effects, reduced spatial migration and non-specific tracer diffusion can impair the discrimination between the tissue of interest and the background. To combat background signals associated with local tracer administration, we explored a pretargeting concept aimed at quenching non-specific fluorescence signals. The efficacy of this concept was evaluated in an *in vivo* neuronal tracing set-up.

**Methods:** Neuronal tracing was achieved using a wheat germ agglutinin (WGA) lectin^.^ functionalized with an azide-containing Cy5 dye (**N_3_-Cy5-WGA**). A Cy7 quencher dye (**Cy7-DBCO**) was subsequently used to yield **Cy7-Cy5-WGA**, a compound wherein the Cy5 emission is quenched by Förster resonance energy transfer to Cy7. The photophysical properties of **N_3_-Cy5-WGA** and **Cy7-Cy5-WGA** were evaluated together with deactivation kinetics *in situ, in vitro* (Schwannoma cell culture)*, ex vivo* (muscle tissue from mice; used for dose optimization), and* in vivo* (*nervus ischiadicus* in THY-1 YFP mice)*.*

**Results:** *In situ*, conjugation of **Cy7-DBCO** to **N_3_-Cy5-WGA** resulted in >90% reduction of the Cy5 fluorescence signal intensity at 30 minutes after addition of the quencher. In cells, pretargeting with the **N_3_-Cy5-WGA** lectin yielded membranous staining, which could efficiently be deactivated by **Cy7-DBCO** over the course of 30 minutes (91% Cy5 signal decrease). In *ex vivo* muscle tissue, administration of **Cy7-DBCO** at the site where **N_3_-Cy5-WGA** was injected induced 80-90% quenching of the Cy5-related signal after 10-20 minutes, while the Cy7-related signal remained stable over time. *In vivo,* **Cy7-DBCO** effectively quenched the non-specific background signal up to 73% within 5 minutes, resulting in a 50% increase in the signal-to-background ratio between the nerve and injection site.

**Conclusion:** The presented pretargeted fluorescence-quenching technology allowed fast and effective reduction of the background signal at the injection site, while preserving *in vivo* nerve visualization. While this proof-of-principle study was focused on imaging of nerves using a fluorescent WGA-lectin, the same concept could in the future also apply to applications such as sentinel node imaging.

## Introduction

Fluorescence-based imaging technologies that enable real-time illumination of specific anatomical structures are rapidly gaining ground in the field of image-guided surgery 1-3. Herein the identification accuracy is highly dependent on the application of fluorescent tracers and accumulation thereof in the tissue of interest. Experimental implementation of new compounds and imaging concepts has been driven by routine clinical application of dyes such as indocyanine green and fluorescein 4. Because light is heavily attenuated in tissue, fluorescence imaging was shown to have a relatively poor *in vivo* sensitivity compared to radio-guided surgery concepts 5, 6. In many cases, this has resulted in the use of high tracer doses 7-9. Reliance on high quantities of tracer not only drives up the cost of these molecular imaging procedures, it also increases the chance of background accumulation 10 that can impair diagnostic accuracy.

When the general location of an imaging target is already well-defined, local tracer administration provides an appealing alternative for intravenous tracer administration. This type of tracer deposition has proven its clinical potential during e.g. sentinel lymph node procedures. Human studies using local tracer administration have even demonstrated that fluorescence guidance can be connected with micro-dosing 5. Alternatively, it has been argued that the up-and-coming concept of nerve imaging can benefit from approaches that rely on local administration to increase the effective local concentration 11 and to minimize the chance of systemic toxic side-effects 12.

A downside of using local administration is the impaired discrimination between the signal diffusing from the injection site and the relatively low signal accumulated in the structures of interest. In radioactivity-based sentinel node imaging procedures, the limitations caused by the background signals are designated as the “shine-through effect”. Here physical shielding of the background signal 13 or image processing of gamma camera images help eliminate the background signal 14, 15. For emerging fluorescence-guided surgery applications similar technologies still need to be developed.

Other than for radioisotope-based imaging, fluorescence imaging allows chemical signal quenching based on distance-dependent energy transfer between fluorophores (< 8 nm distance between dye molecules). This Förster resonance energy transfer (FRET) phenomenon is used to excite one fluorophore with the emission of another 16, 17. FRET can also be employed as a means of silencing the emission with the lowest wavelength, a concept that is widely explored in the design of activatable fluorescent tracers 18. Specific FRET-based fluorescence deactivation was recently also explored as diagnostic read-out during receptor-(pre)targeted theranostics *in vitro* 19.

**Scheme 1:**
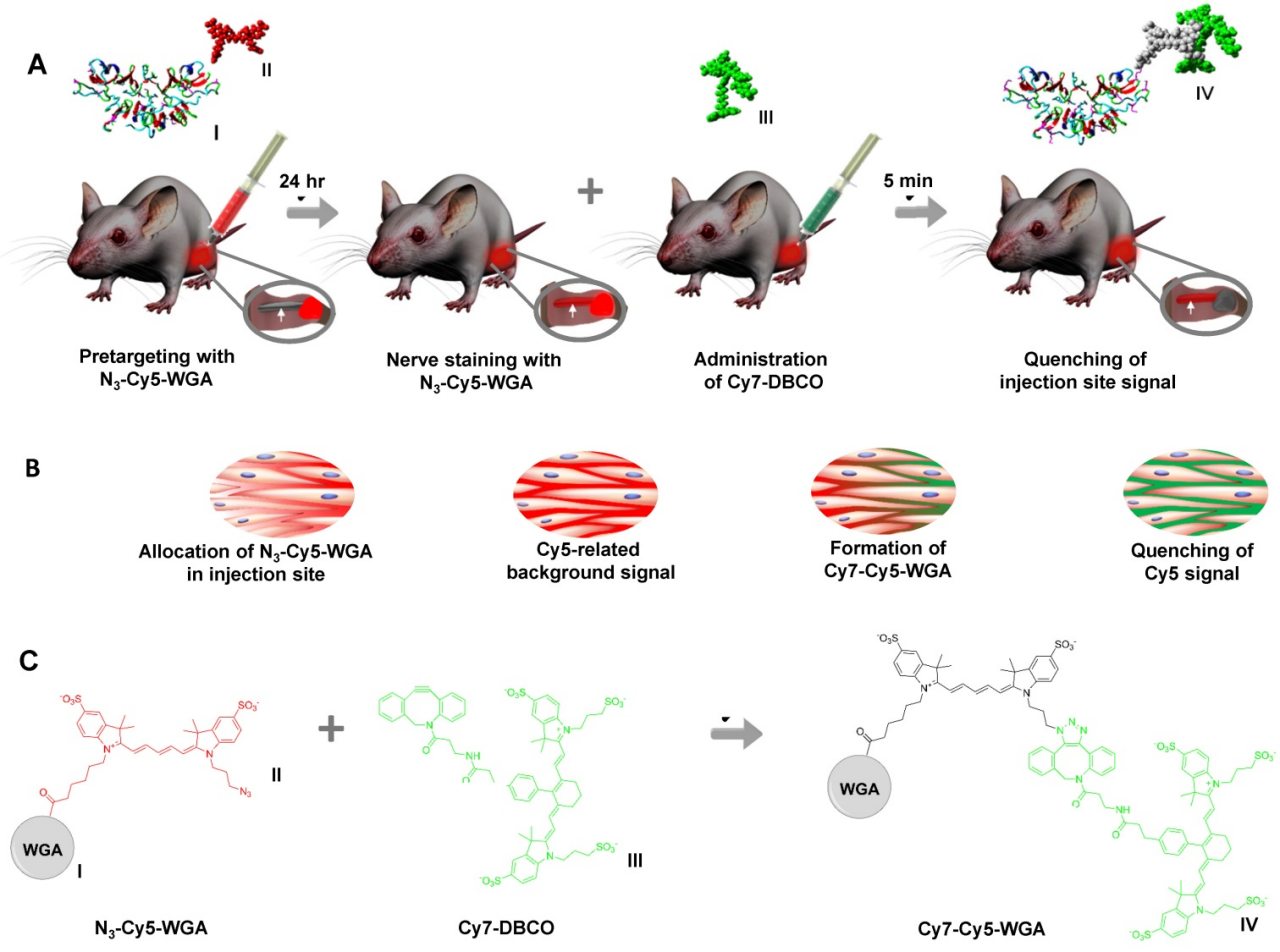
** Schematic overview of deactivation signal injection site. A)** Molecular representation of **N_3_-Cy5-WGA** with I representing WGA and II representing N_3_-Cy5. Pretargeting through injection of **N_3_-Cy5-WGA** (in red) in the *quadriceps femoris* muscle near the *nervus ischiadicus* and nerve staining at 24 hours after tracer administration. Insert showing the *nervus ischiadicus* (white arrow) and the injection site (in red) located in the hind leg. Molecular representation of **Cy7-DBCO** (III) and injection of **Cy7-DBCO** (in green) followed by subsequent Cy5-quenching at the injection site within 5 minutes after quencher administration. Insert showing the portion of the *nervus ischiadicus* stained by **N_3_-Cy5-WGA** (in red) and the quenching of the primary injection site (in grey) after the formation of **Cy7-Cy5-WGA** (IV). **B)** The corresponding distribution of **N_3_-Cy5-WGA** (Cy5 in red) and** DBCO-Cy7/Cy7-Cy5-DBCO** (Cy7 in green) over the extracellular space in the muscle tissue of the injection site during the different stages in the process as described in A. **C)** Reaction scheme including chemical structures of **N_3_-Cy5-WGA, Cy7-DBCO,** and **Cy7-Cy5-WGA.**

Here, we set out to determine if FRET-based fluorescence deactivation could be of value for *in vivo* imaging applications. Specifically, the ability to use fluorescence deactivation for background reduction during neuronal tracing of the *nervus ischiadicus* was studied using an azide-Cy5 functionalized analog of the known neuronal tracer wheat germ agglutinin (WGA) lectin (**N_3_-Cy5-WGA**; Scheme 1) 11; WGA has an affinity for proteoglycans present on the extracellular matrix of peripheral nerves 20, allowing labeling without disrupting signal conduction within the nerve 11, 21. Following neuronal pretargeting using **N_3_-Cy5-WGA**, fluorescence deactivation of the residual neuronal tracer at the injection site was realized via FRET quenching following click-chemistry-based conjugation of **Cy7-DBCO** (Scheme 1A/B). The quenching mechanism was first studied *in situ* and* in vitro,* followed by feasibility studies* ex vivo* in muscle tissue and* in vivo* in mice.

## Materials and Methods

### Synthesis N_3_-Cy5-OSu

**N_3_-Cy5** was synthesized according to previously described methods 19, using an on-resin method as described by Lopalco et al 22. Cleavage of the dye was achieved by the addition of (SO_3_)-indole phthalimide to resin-immobilized hemicyanine. After purification by semi-preparative HPLC (Waters 1525EF, Waters 2489, Reprosil-Pur 120 C18-AQ 250 x 10 mm), the amine group was liberated using methylamine and converted to an azide followed by HPLC-purification and lyophilization. Of the resulting blue solid, 5 mg (7.03 µmol) was dissolved in DMSO (200 µL). To this solution, Dipyrrolidino(N-succinimidyloxy)carbenium hexafluorophosphate (6 mg, 14.07 µmol) and N-methylmorpholine (3.9 µL, 35.16 µmol) were added. The mixture was stirred for 45 minutes at room temperature, after which CH_3_CN containing 0.1% trifluoroacetic acid (300 µL) and H_2_O containing 0.1% trifluoroacetic acid (1500 µL) were added followed by purification using HPLC. The fractions containing N_3_-Cy5-OSu were combined and lyophilized, yielding a blue powder (4.5 mg, 79% yield).

### Synthesis N_3_-Cy5-WGA

WGA (1 mg, 27.8 µmol) was dissolved in 0.1 M phosphate buffer pH 8.4 (200 µL), where after 138.9 µmol of N_3_-Cy5-OSu (11.2 µL of a 10 µg/µL solution, in DMSO) was added. The mixture was stirred for 2 hours at room temperature before being purified using a 10 K Amicon centrifugal filter (Merck KGaA, Darmstadt, Germany). After repetitive washing until a colorless filtrate was observed, N_3_-Cy5-WGA was recovered as a blue liquid (20% yield).

### Evaluation photophysical properties

The molar extinction coefficient and relative quantum Yield (ɸ_F_) were determined according to previously described methods 19, 23. A dilution range was made in PBS (0.01 M, pH 7.4) using a quantified amount of **N_3_-Cy5** (either by weighing or quantitative NMR). The absorption of the dilution series was measured, and the maximum absorption values were plotted against the concentration and the molar absorption coefficient was calculated using linear regression. The quantum yields were determined by measuring absorbance and emission of 3-6 dilutions of the compound, thereby keeping the absorbance wavelength of interest below 0.100 Abs. The absorbance at one wavelength was plotted against the total emission by excitation at this same wavelength. The quantum yield was thereafter calculated using linear regression relative to that of a reference dye which was measured simultaneously. Emission spectra were extracted from the recorded epsilon and quantum yield data. The stability of **N_3_-Cy5-WGA** was determined using absorption measurements after 24 hours of incubation at 37°C 23. *In situ* quenching was assessed after addition of two molar equivalents of **Cy7-DBCO** to 3 mL of a 1 µM solution of **N_3_-Cy5-WGA** or **Cy5-NH_2_** (non-azide-containing control; dye synthesis described in 19). The change in fluorescence over time was evaluated using repeated measurement of the emission spectrum (60 minutes with a 5-minute interval between measurements using excitation light of 620 nm and an excitation- and emission slit of 10), as described previously 19.

### Fluorescence confocal imaging of cells

RT4 D6P2T Schwannoma cells (CRL-2768, ATCC) were cultured in Dulbecco's Modified Eagle Medium (Life Technologies, UK) containing penicillin, streptomycin and fetal calf serum (All BD Biosciences) at 37°C and 5% CO_2_. Two days prior to imaging, cells were seeded onto glass-bottom culture dishes (MatTek). At one hour prior to imaging, **N_3_-Cy5-WGA** (5 µL/0.58 nmol) was added to the medium and incubated at 4°C. A lysosomal (lysotracker green; 2 µL/mL, DND-26, Thermo Fisher) and nuclear stain (Hoechst 33342; 1 mg/mL, Thermo Fisher) was added as intracellular reference staining. Cell samples were washed thrice with PBS and placed on a Leica SP8 WLL confocal microscope (Leica Microsystems) for imaging. Images were acquired prior to addition of **Cy7-DBCO** (t=0; 800 μM in DMSO stock, 1 μl/ 0.8 nmol per sample resulting in a ratio of 10:14 Cy5:Cy7; 19) and over the course of 30 minutes after addition of **Cy7-DBCO** to the medium (at room temperature) using Leica confocal software (Leica Microsystems) at sequential settings and 63x magnification (Cy5 settings for visualization of **N_3_-Cy5-WGA**: λ_ex_ 633 nm, ; λ_em_ 650-700 nm; FITC settings for visualization of lysosomal staining: λ_ex_ 488, λ_em_ 500-550 nm; UV settings for visualization of nuclear staining: λ_ex_ 405 nm, λ_em_ 420-452 nm). Quantification of the Cy5 signal was performed using the quantification tool in Fiji software according to previously described methods 19. For calculating normalized fluorescence intensities per time point, t=0 (prior to addition of **Cy7-DCO**) was set at 100%.

### *Ex vivo* and *in vivo* quenching of the injection site

Muscle tissue was obtained from THY-1 YFP mice (n = 6 muscle specimens (*quadriceps femoris* muscle) per group). For assessment of signal quenching, muscle tissue specimens were imaged using an IVIS Spectrum preclinical imaging system (Perkin Elmer) and Living Image software (version 3.2). Prior to imaging, samples were superficially injected with **N_3_-Cy5-WGA** (5 µl, 0.58 nmol). For optimization of the volume/dose of **Cy7-DBCO** required for optimal quenching in tissue, samples were then either injected with 5, 10, or 20 µl (6.6-26.6 nmol resulting in ratios Cy5:Cy7 varying from 10:114 to 10:458) **Cy7-DBCO.** Tissue samples wherein only **N_3_-Cy5-WGA** was administered were used as a control. Tissue samples were imaged at Cy5 (λ_ex_ 650 nm, λ_em_ 700 nm) and Cy7 (λ_ex_ 780 nm, λ_em_ 800 nm) settings prior to addition of **Cy7-DBCO** (t=0) and followed over time (for 30 minutes at room temperature) after addition of the quencher.

The selection of the *nervus ischiadicus* was based on its accessible location and the possibility to reproducibly place tracer deposits in the *quadriceps femoris* muscle and subsequently assess staining of the nerve. Neuronal tracing of the *nervus ischiadicus* was assessed according to previously described methods 11.* In vivo* assessment of signal quenching was evaluated in THY-1 YFP mice (n = 4 received both **N_3_-Cy5-WGA** and **Cy7-DBCO**, n = 2 served as control (only **N_3_-Cy5-WGA**, no** Cy7-DBCO**) at 24 hours after intramuscular injection of 20 µl (2.32 nmol) **N_3_-Cy5-WGA** allowing migration of the tracer along the *nervus ischiadicus* 11. Prior to imaging mice were sacrificed and the *quadriceps femoris* muscle and *nervus ischiadicus* were exposed. **Cy7-DBCO** (10 µl,13.2 nmol, resulting in a 10:57 ratio for Cy5:Cy7) was injected into the injection site of **N_3_-Cy5-WGA** and images were obtained prior and at 5 minutes after injection of **Cy7-DBCO** using an IVIS Spectrum imaging system (Xenogen) and using a Dino-lite handheld digital fluorescence microscope (AM4115T-DFRW for Cy5 imaging; Dino-lite Digital Microscope).

Signal intensities in the individual samples were quantified (per ROI in photons/sec/cm^2^) using the quantification tool in the Living Image software 11. For comparison between groups, fluorescence signals were normalized; here t=0 was set at 100%.

The area of distribution of the quencher over the injection site and the signal-to-background ratio (SBR; signal in the nerve signal/ signal injection site) were evaluated based on the IVIS images, using the ROI measuring tool in Fiji software. Distribution of the Cy7 signal over the muscle specimen was calculated as a percentage of the total area of the muscle specimen. Statistical analysis was performed using a Student's t-test and the level of significance was set at *p* < 0.05.

## Results

### Photophysical properties

**Figure 1:**
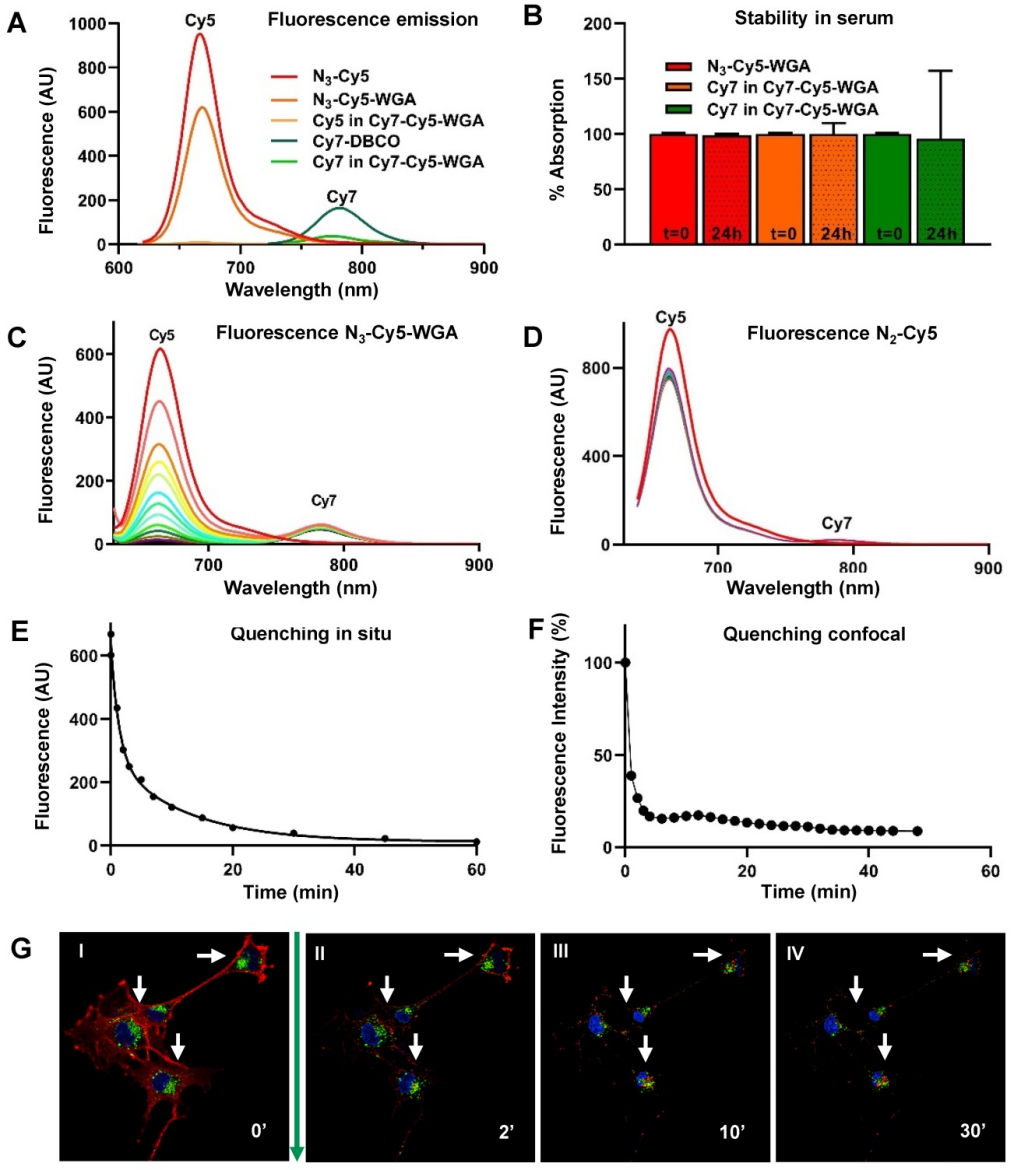
***In situ* and *in vitro* quenching of N_3_-Cy5-WGA after addition of Cy7-DBCO. A)** Fluorescence absorption and emission of **N_3_-Cy5, N_3_-Cy5-WGA**, **Cy7-DBCO,** and the Cy5 and Cy7 signal in **Cy7-Cy5-WGA**. **B)** Stability of **N_3_-Cy5-WGA** and the Cy5 and Cy7 signal in **Cy7-Cy5** in serum at t = 0 and t = 24 hours (dashed). Absorbance spectroscopy over the course of 60 minutes with a 5-minute interval between measurements (color coding per interval from red to purple) of the absorbance spectrum of **C) N_3_-Cy5-WGA** and **D)** the non-azide containing **Cy5-NH_2_** after mixing with **Cy7-DBCO** (excitation light of 620 nm and an excitation- and emission slit of 10) with color scaling between t=0 (highest Cy5 intensity) and t=60 minutes.** N_3_-Cy5-WGA** emission peak detected at 630 nm, **Cy7-DBCO** emission peak detected at 780 nm. **E)** Normalized change of *in situ* fluorescence intensity over time after addition of **Cy7-DBCO** to **N_3_-Cy5-WGA**. **F)** Quantified decrease of *in vitro* **N_3_-Cy5-WGA**-related fluorescence in RT4 D6P2T cells assessed using fluorescence confocal microscopy over time after addition of** Cy7-DBCO** and subsequent formation of **Cy7-Cy5-WGA**. Herein the fluorescence intensity (%) depicts the normalized fluorescence percentage of fluorescence intensity, wherein the intensity measured at t = 0 (prior to addition of **Cy7-DBCO**) was set at 100%. **G)** Fluorescence confocal microscopy images of RT4 D6P2T cells after addition of **N_3_-Cy5-WGA** (I; t = 0 minutes, λ_ex_ 633 nm) and at II) t = 1 minutes, III) t = 5 minutes and IV) t = 30 minutes after addition of **Cy7-DBCO** with **N_3_-Cy5-WGA** in red, lysosomes in green and the cell nucleus in blue.

The photophysical properties of the individual dyes **N_3_-Cy5** and **Cy7-DBCO,** as well as the **N_3_-Cy5-WGA** and **Cy7-Cy5-WGA** constructs, are listed in Table 1. Accompanying fluorescence and absorption spectra are provided in Figure 1A. The emission wavelength of **N_3_-Cy5-WGA** was shown to be almost identical to that of free **N_3_-Cy5**, while the brightness of **N_3_-Cy5-WGA** was slightly decreased compared to the free dye. Interestingly, the formation of the **Cy7-Cy5** construct caused the Cy5 excitation peak to undergo a hypsochromic shift of 27 nm and the Cy7 emission peak to undergo a hypsochromic shift of 24 nm. Also, the relative quantum yields of Cy5 and Cy7 decreased respectively 36-fold and 7.7-fold compared to the free dyes. Since the molar extinction coefficient of Cy7 also decreased 6-fold after conjugation, the Cy7 brightness in the **Cy7-Cy5** construct dropped 50-fold.

Stability measurements **of N_3_-Cy5-WGA**, **Cy5,** and **Cy7** in the** Cy7-Cy5** construct in serum revealed that the dye-protein combination remained stable in serum (100%, 99%, and 96%, respectively) over the course of 24 hours (Figure 1B).

*In situ*, conjugation of **Cy7-DBCO** to **N_3_-Cy5-WGA** yielded** Cy7-Cy5-WGA** (Scheme 1), with subsequent quenching of 98.1% of the overall Cy5 signal and reduced brightness for Cy7 (Figure 1C; Cy5 peak at 650 nm, Cy7 peak at 780 nm). Negligible quenching was observed between a non-azide containing Cy5 derivative (**Cy5-NH_2_**) and **Cy7-DBCO** (Figure 1D). Repeated measurements of the absorbance spectrum of **N_3_-Cy5-WGA** revealed a sharp decline in signal intensity within minutes after addition of **Cy7-DBCO** and an overall signal reduction of >90% of the signal intensity of Cy5 fluorescence over the course of 60 minutes (Figures 1A and 1E)**.**

Hereafter, the deactivatable concept was studied *in vitro* using Schwannoma cell cultures (Figure 1F and 1G). In cells, **N_3_-Cy5-WGA** enabled a clear definition of the cell membrane (Figure 1G I), which could clearly be differentiated from the co-staining of lysosomes (in green) and the nucleus (in blue). After addition of **Cy7-DBCO** and formation of** Cy7-Cy5-WGA**, the staining of the cell membrane was shown to fade over time (Figure 1G II-IV), while the localization of nuclear and lysosomal control staining was not affected. When quantified, a decrease in signal intensity ranging from 61.5% (t = 0) to 91% (t = 30 minutes), after addition of **Cy7-DBCO**, was recorded. Subsequently, the level of signal quenching remained stable.

### Quenching in muscle tissue

**Figure 2:**
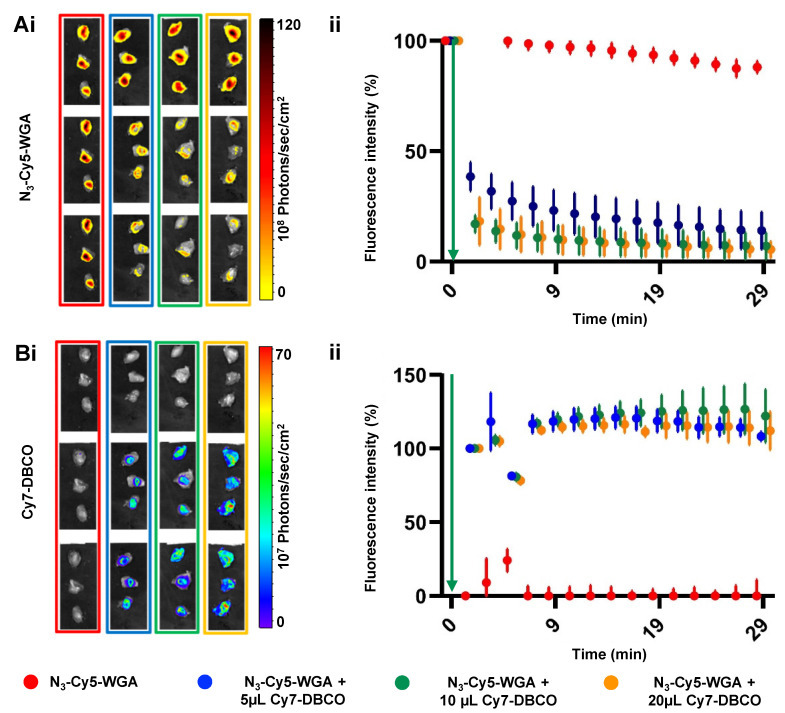
** Quantification of deactivation signal in muscle tissue.** Ai) Overlay of *ex vivo* Cy5-related fluorescence images (λ_ex_ 650 nm) and a photograph of muscle tissue specimens after injection of 5 μL **N_3_-Cy5-WGA** alone (no **Cy7-DBCO**; in red) 5 μL **N_3_-Cy5-WGA** and at t = 1, t= 5 and t = 30 minutes after addition of 5 (blue), 10 (green) or 20 μL (orange) of **Cy7-DBCO**. ii) Quantified Cy5-related signal intensities in muscle tissue specimens per group over time (t = 0-30 minutes). Green arrow: time point of addition of **Cy7-DBCO**. Bi) Overlay of *ex vivo* Cy7-related fluorescence images (λ_ex_ 780 nm) and a photograph of muscle tissue specimens without **Cy7-DBCO** (red) and after injection of 5 (blue), 10 (green) or 20 μL (orange) **Cy7-DBCO**. Green arrow: time point of addition of **Cy7-DBCO.** ii) Quantified Cy7-related signal intensities in the muscle tissue specimens per group over time (t = 0-30 minutes).

Local injection of **N_3_-Cy5-WGA** (20 μL, 2.32 nmol) in the *triceps brachii* muscle provides a model to study the deactivation concept in tissue (Figure 2). Based on the *in situ* and *in vitro* data (Figure 1), *ex vivo* assessment of the quenching of the injection site was performed over the course of 30 minutes. Under these conditions injection of **Cy7-DBCO** into muscle tissue allowed for the click reaction to occur, thus yielding a decrease in signal intensity as a result of FRET quenching (Figure 2; WGA alone in red). Increasing the volume of **Cy7-DBCO** (Figure 2; from 5 μL (6.6 nmol; 11 molar equivalents to Cy5; in blue) to 10 μL (13.3 nmol; 23 molar equivalents to Cy5; in green) and 20 μL (25.6 nmol; 46 molar equivalents to Cy5; in orange) increased the interstitial fluid pressure. The use of the different volumes resulted in different levels of Cy7 diffusion in the muscle tissue specimens; immediately after administration of 5 μL of **Cy7-DBCO** a tissue coverage of 46.2 ± 7.1% was reached, whereas after administration of 10 μL or 20 μL the Cy7 signal was shown to be distributed over respectively 73.7 ± 2.8% and 77.3 ± 14.3%, of the tissue specimen. At 20 minutes post administration of **Cy7-DBCO** the tissue coverage was not significantly altered (45.5 ± 8.1%, 77.7 ± 8.1% and 78.1 ± 13.2% for 5 μL, 10 μL, and 20 μL, respectively).

The distribution of **Cy7-DBCO** impacted on the quenched portion of **N_3_-Cy5-WGA**; an immediate decrease in signal intensity was seen in all samples wherein **Cy7-DBCO** was administered ranging from 62.5% for the 5 μL group to 82.8% and 82.7% for the 10 μL and 20 μL groups, respectively (Figure 2Ai and 2Bi). Quantification of the Cy5 signal in the tissue samples confirmed the visible quenching effects (Figure 2A and B). The signal decrease intensified until 20 minutes post administration of **Cy7-DBCO** (to respectively 76.8%, 89.9% and 90.1% for 5 μL, 10 μL, and 20 μL at 10 minutes and 82.4%, 91.7% and 93% for 5 μL, 10 μL, and 20 μL at 20 minutes), where after the signal remained relatively stable (86%, 92.9% and 94.5% decrease in fluorescence signal for the 5 μL, 10 μL and 20 μL groups at 30 minutes). In contrast, the fluorescence signal in the **N_3_-Cy5-WGA** group (no **Cy7-DBCO**) only showed a slight decrease of 12%, which could possibly be attributed to dye bleaching as a result of the repetitive imaging of the same sample. The Cy7 signal emitted by **Cy7-DBCO,** and subsequently by **Cy7-Cy5-WGA,** could be accurately visualized upon tracer administration (Figure 2Bi) and remained relatively stable over time (Figure 2Bii).

### Nerve tracing and *in vivo* quenching of background signal

**Figure 3:**
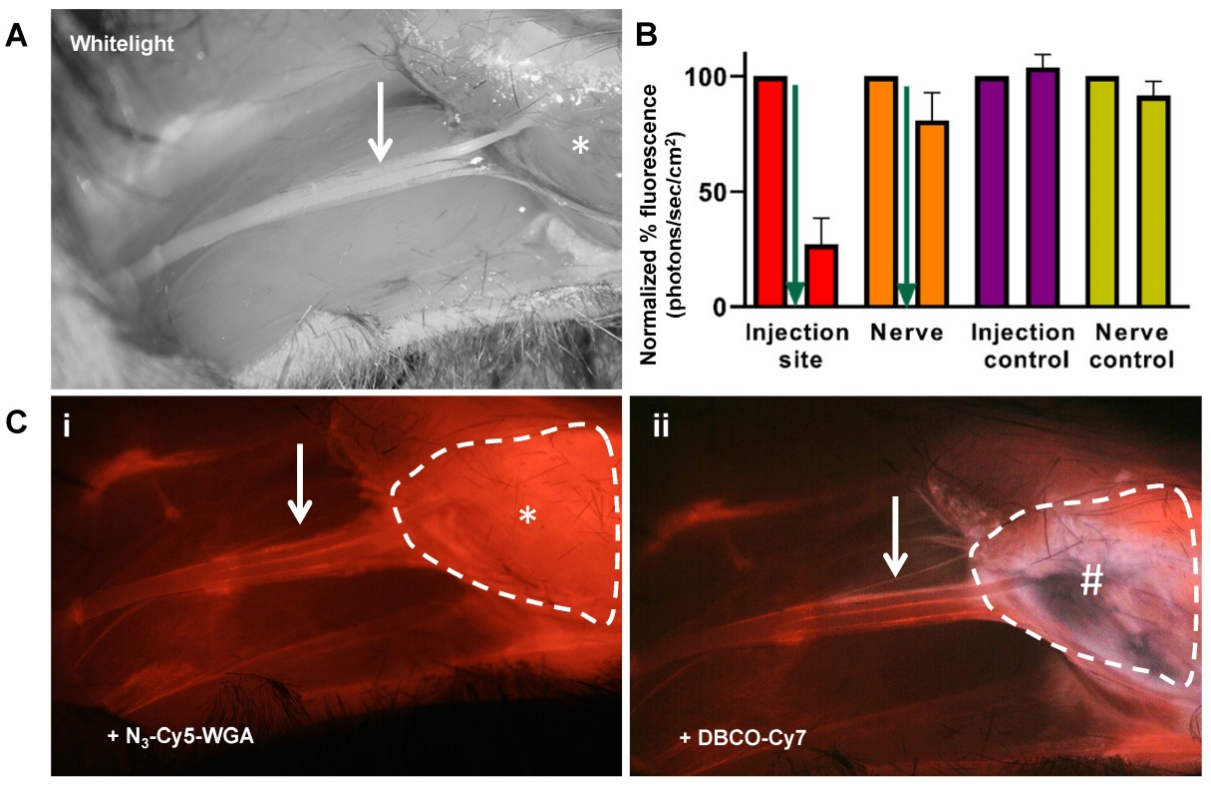
** Quenching of the injection site *in vivo*.** A) *In vivo* white-light image of the *nervus ischiadicus* (white arrow) and its anatomical localization (*: location of tracer deposition). B) Comparison of the quantified *in vivo* percentage of Cy5-related fluorescence (λ_ex_ 650 nm) measured in the injection site (red) and nerve (orange) at 24 hours after injection of **N_3_-Cy5-WGA** (left bar) and 5 minutes after injection of **Cy7-DBCO** (right bar). Administration of **Cy7-DBCO**: green arrow. Animals wherein only **N_3_-Cy5-WGA** was administered served as control. C) i) fluorescence microscope image showing the injection site (* and encircled in white) and the *nervus ischiadicus*. ii) fluorescence microscope image after injection of **Cy7-DBCO** (#) at the site of injection of **N_3_-Cy5-WGA**.

*In vivo* administration of **N_3_-Cy5-WGA** allowed for distinct visualization of tracer migration along the *nervus ischiadicus* (Figure 3) and quantitative assessment of the quenching effect at the site injection (Figure 3B). Initially, an accurate assessment of the stained portion of the nerve (Figure 3, white arrow) was hampered by an intense background signal emitted by the intramuscular deposition site of **N_3_-Cy5-WGA** (Figure 3Ci; *, encircled). Administration of **Cy7-DBCO** (Figure 3B, green arrow) resulted in a fast and significant 73% depletion of Cy5-related background signal within the first 5 minutes. While a slight decrease in signal was also seen in the nerve (19%), a clear distinction between the injection site and the nerve could be made (Figure 3B and 3Cii). Overall, quenching resulted in a 2-fold increase in the SBR between the nerve and the injection site (1.0 ± 0.2 vs. 2.1 ± 0.5; *p*= 0.03).

## Discussion

In the current manuscript, we have demonstrated that fluorescence deactivation could be successfully translated from *in situ* experiments to an *in vivo* application relying on local tracer deposition. Hereby, we have essentially introduced a fluorescence deactivation paradigm based on a pretargeting concept wherein the signal from non-specifically diffused fractions of primary targeting vector can be actively silenced using a secondary compound.

In line with prior literature 19, 25 the current work illustrates that orthogonal “click” chemistry provides a valuable chemical tool in the conjugation of (quencher) dyes to the primary fluorescent agent. In particular, the ability to swiftly conjugate **Cy7-DBCO** to** N_3_-Cy5** ensures that both dyes are in close vicinity of each other (< 8 nm) and FRET transfer between the dyes is optimal as the dyes are both residing on the same molecule, hence the high degree, and rapid quenching, of the Cy5 signal of **N_3_-Cy5** and not for** Cy5-NH_2_** (Figures 1A and 1C). The quenching of Cy7 as well as the differences in quantum yields and molar extinction coefficient can most likely be ascribed to π-stacking 24, although cis-trans photoisomerization - an effect increased by steric hindrance- could also play a role 26, 27.

Pretargeting strategies are known to use a range of alternative forms of specific recognition of the primary targeting vector, e.g. based on monoclonal antibodies 28, 29 or host-guest chemistry 30. In theory, these alternative means of connecting the secondary quenching agent to the fluorescent primary vector could also hold potential for fluorescence quenching applications. That said, it is likely that the increased spatial separation of the dyes in these indications will reduce quenching efficacy; FRET is distance dependent.

In a previous microscopy study, we showed that **Cy7-DBCO** does not have the ability to pass the cell membrane at 4 °C or 37 °C 19, and thus merely diffuses through the tissue. It is important to note that **Cy7-DBCO** could only realize quenching by formation of a covalent bond with **N_3_-Cy5-WGA** (Figure 1). Hence, the **Cy7-DBCO** diffusion, combined with the need for covalent bonds and the neuronal tracing capabilities of **N_3_-Cy5-WGA** 11, suggests that the 2-fold increase in the SBR observed following* in vivo* addition of **Cy7-DBCO** (Figure 2 and 3) is the result of **Cy7-Cy5-WGA** formation in the interstitial space. This efficient use of dye-based click-chemistry extends previous *in vitro* findings 19.

While the fluorescence deactivation concept may also prove of value during systemic applications, it is likely to hold most promise in procedures were local tracer deposition is being used. Next to experimental nerve imaging 31, 32, this could e.g. be embolization strategies 33, or sentinel lymph node procedures 6. A limitation of implementing the fluorescence deactivation concept and the enabling chemical strategies used is the cost. For example, a routine sentinel node procedure, while clearly benefiting from techniques that reduce background staining 34, is not likely to warrant the development and approval costs of new pharmaceuticals for this indication. For more specialized indications, such as nerve imaging, this will likely be different.

## Conclusion

The presented fluorescence deactivation technology allowed fast and effective reduction of the background signal at the injection site while preserving *in vivo* nerve visualization. While this proof-of-principle study was focused on imaging of nerves, it helps create a new fluorescence imaging paradigm.

## Figures and Tables

**Table 1:** Photophysical properties **N_3_-Cy5** and **Cy7-DBCO**

Tracer	Abs/Em in PBS (Stokes shift in nm)	Relative quantum yield (in PBS)	Molar extinction coefficient in PBS (L*Mol^-1^*cm^-1^)	Brightness
N_3_-Cy5	648/666 (18)	26.0%*	187 600	48 776
N_3_-Cy5-WGA	649/667 (19)	18.0%	n.d.	33 768^¥^
Cy7-DBCO	753/775 (22)**	10.8%	255 000**	28 050
Cy5 in Cy7-Cy5 construct	620/666 (46)	0.5%	185 000	925
Cy7 in Cy7-Cy5 construct	799/775 (24)	1.4%	39 900	559

*previously reported by van der Wal et al. 24 **as provided by the manufacturer, ¥: Calculated using the molar extinction coefficient of the free dye, n.d.: not determined.
